# Comparison of hypothalamo-pituitary-adrenal function in treatment resistant unipolar and bipolar depression

**DOI:** 10.1038/s41398-021-01343-5

**Published:** 2021-04-26

**Authors:** Kalypso Markopoulou, Susanne Fischer, Andrew Papadopoulos, Lucia Poon, Lena J. Rane, Abebaw Fekadu, Anthony J. Cleare

**Affiliations:** 1 Centre for Affective Disorders, Institute of Psychiatry, Psychology & Neuroscience, King’s College London, London, UK; 2 National Institute for Health Research (NIHR) Mental Health Biomedical Research Centre, South London and Maudsley NHS Foundation Trust and King’s College London, London, UK; 3 Affective Disorders Unit and Laboratory, South London and Maudsley NHS Foundation Trust, London, UK; 4 Clinical Psychology and Psychotherapy, Institute of Psychology, University of Zurich, Zürich, Switzerland; 5 Centre for Innovative Drug Development and Therapeutic Trials for Africa, College of Health Sciences, Addis Ababa University, Addis Ababa, Ethiopia

**Keywords:** Diagnostic markers, Physiology

## Abstract

Altered functioning of the hypothalamic-pituitary-adrenal (HPA) axis has been demonstrated in patients with treatment-resistant depression, although studies have often conflated patients with unipolar and bipolar depression. This is problematic given that the two groups often present with opposed neurovegetative symptom patterns. The aim of this study was to test, for the first time, whether post-awakening cortisol, a highly reliable, naturalistic measure of HPA functioning, could distinguish patients with clearly defined treatment-resistant unipolar (TRUD) and bipolar depression (TRBD). A total of 37 patients with TRUD, 17 patients with TRBD, and 47 healthy controls were recruited. Areas under the curve (AUC) with respect to the ground (g) and increase (i) of post-awakening cortisol concentrations (awakening, +15, +30, +45, +60, +90 min) were measured over two days. Patients with TRUD had higher total cortisol production in the morning hours compared to controls (AUCg, *p* = 0.01), while they did not differ in terms of the awakening response (AUCi, *p* = 0.28). By contrast, subjects with TRBD had lower total cortisol when compared to controls by trend (AUCg, *p* = 0.07), while they did not differ in the awakening response (AUCi, *p* = 0.15). A direct comparison of TRUD and TRBD revealed differences in the AUCg (*p* = 0.003) and AUCi (*p* = 0.03). This finding of comparatively elevated HPA axis activity in the morning in TRUD and attenuated HPA axis activity in TRBD attests to a fundamental biological distinction between unipolar and bipolar depression. It has implications for the understanding and treatment of bipolar depression and in differentiating the two types of depression.

## Introduction

Depression is a debilitating condition, with a course that is often recurrent or chronic with a negative impact on both society and the individual^1^. Its high prevalence and the associated functional impairment make depression a major cause of disease burden around the world^2^; due to its longer duration and severity, treatment-resistant depression significantly contributes to the above burden^3^.

Although there has been extensive research into the link between hypothalamic-pituitary-adrenal (HPA) axis abnormalities and unipolar depression, with findings such as elevated basal cortisol levels, non-suppression of cortisol following dexamethasone administration, and alterations in the cortisol awakening response^4,5^, research looking into depressive episodes occurring as part of bipolar disorder—bipolar depression—has been much less extensive^6^. This is especially true regarding post-awakening cortisol concentrations, with only three published studies comparing patients with bipolar depression to healthy controls, two reporting null-findings^7,8^ and one reporting higher levels in patients^9^.

Matters are complicated further by the likelihood that in some studies, investigators have combined individuals with unipolar and bipolar depression within one illness group, thus confounding results and masking any potential differences between these two forms of depression. Certain clinical features, such as hypersomnia, diminished energy levels, diurnal mood variation, psychosis, excessive self-reproach, and low libido are more common in bipolar rather than unipolar depression^10^. Interestingly, several of these characteristics are also encountered in atypical depression, which is characterised by reversed neurovegetative symptoms and seems to exhibit a different endocrine profile compared to melancholic depression, namely hypocortisolemia as opposed to hypercortisolemia^11^.

In addition to this diagnostic issue, very few studies to date have differentiated or attempted to measure the above biological parameters in clearly defined treatment-resistant affective disorders. Studies in unipolar depression have suggested that hypercortisolemia is a factor associated with treatment resistance^12–14^, but to date, there have been no studies in treatment-resistant bipolar depression.

One of the most relevant HPA axis indices is post-awakening cortisol concentrations, including the so-called cortisol awakening response (CAR). Although its function still remains unclear, perhaps relating to the anticipation of the demands of the day ahead^15^, the CAR has been extensively used as a measure of HPA axis reactivity to a natural challenge^16^. However, despite offering a highly reliable, naturalistic way of assessing both HPA axis activity and reactivity, post-awakening cortisol levels have only been measured once in a clearly defined population of treatment-resistant depression^17^, showing no abnormalities when compared to matched healthy controls. Notably, in this study the focus was on ‘major depressive episodes’, hence there was no explicit distinction between treatment resistant unipolar (TRUD) and bipolar depression (TRBD).

The purpose of the present study was therefore to investigate HPA axis functioning in treatment-resistant depression, and specifically to compare unipolar and bipolar depression, using post-awakening cortisol concentrations, including the CAR.

## Methods and materials

### Participants

Recruitment of participants aged 18–75 with TRUD and TRBD was undertaken through the National Affective Disorders Services, at the South London and Maudsley (SLAM) NHS Foundation Trust, a tertiary care service for treatment-resistant affective disorders. Patients were predominantly current or previous inpatients in the service, with a minority recruited exclusively from the outpatient service (notably, the two patient groups did not differ in terms of inpatient vs. outpatient status). All patients met the criteria for a current depressive episode using the 10th edition of the International Classification of Diseases (ICD-10). Patients were included in the unipolar depression group if they met criteria for recurrent depressive disorder or a single depressive episode, and into the bipolar depression group if they met criteria for bipolar affective disorder, currently depressed. Diagnoses were made by consensus following a longitudinal psychiatric assessment by at least two psychiatrists supplemented by the Mini International Neuropsychiatric Interview^18^. The 21 item version of the Hamilton Rating Scale for Depression (HAMD 21) was used to assess the severity of the current depressive episode^19^. Only patients with a score of at least 17 points were included. To define treatment resistance, patients needed as a minimum to have failed to respond to at least two classes of antidepressants at the minimum therapeutic dose and duration^20,21^. This is the most widely used definition of treatment resistance; whilst duration of depression is not a part of the definition, in practice the large majority of those with a single depressive episode also had chronic depression (i.e. episode duration of 2 years or more). Given that there is no widely accepted definition of TRBD, the same criteria as for TRUD were used, although other criteria also including mood stabiliser use have more recently been proposed for TRBD^22^. General exclusion criteria were: an organic cause for depression; a systemic physical illness or neurological condition that affects cortisol levels; regular use of corticosteroids or other medication affecting the HPA axis; heavy smoking (>20 cigarettes per day); alcohol dependence or recreational drug use in the last 6 months; and pregnancy or lactation. Neither comorbid mental disorders nor being on psychotropic medication were exclusionary criteria, but both were recorded.

Controls aged 18–75 years were recruited from our database of volunteers, which included hospital and university staff and students and also members of the local community. Exclusionary criteria for controls included a personal history of any form of mental disorder; a history of mental disorder in a first-degree relative; any current physical or systemic illness; use of any medication likely to affect the HPA axis; a current Beck Depression Inventory^23^ score >10; and a positive urine drug screen. Controls were matched with patients according to their age ± 5 years, sex, and Body Mass Index (BMI) ± 5 kg/m^2^.

Applying the eligibility criteria resulted in three groups: *n* = 37 individuals with TRUD, *n* = 17 individuals with TRBD, and *n* = 47 healthy controls. Although the sample sizes of the three groups were not specifically calculated for the purpose of the present study, the number of recruited individuals is similar to previous research on post-awakening cortisol in individuals with depression^6,24^.

The research protocol was approved by the local research ethics committee (London: Camberwell St Giles) and written informed consent was obtained from all participants after receiving a complete description of the study.

### Cortisol measurement

Two measures of post-awakening cortisol were of interest in the present study: (1) total cortisol production in the morning hours, and (2) the CAR. Participants were asked to provide six salivary samples in the morning (upon awakening, and +15, +30, +45, +60, and +90 min thereafter) over two consecutive days, which could be any weekday excluding Monday. They were provided with oral and written instructions, which explained in simple terms the scientific reasons for undertaking the study and how the sampling was to be done. Participants were to try and avoid undertaking any strenuous leisure activities, late nights, and use of alcohol during the two collection days. They were not to have anything to eat or drink or brush their teeth until after the 90 min of sampling. Samples were collected in polypropylene tubes using the passive drool method. Inpatients were instructed to hand in the daily samples to a member of staff and outpatients were provided with an envelope to post the samples back to the Affective Disorders Unit laboratory after storing in a refrigerator in the interim. All samples were stored at −40 °C and cortisol concentrations were determined using the luminescence assay of ‘Immulite’—Siemens’ automated Immunoassay analyser (www.diagnostic.siemens.com), as described in Mondelli et al.^25^. Inter- and intra-assay variance was <10%.

### Statistical analysis

Post-awakening cortisol levels were measured by calculating the areas under the curve with respect to the ground (AUCg) and with respect to the increase (AUCi)^26^. The former is thought to reflect the total cortisol production in the morning hours and the latter indicates the CAR. Compliance was defined as the sample being recorded as taken within 10 min of the scheduled time. In those cases, where there were missing values other than at 0 and 90 mins, the mean value of the two proximal time points was inserted. Where either 0 or 90 min values were missing, the AUC was not calculated. We calculated Pearson’s product–moment coefficients, Spearman rank correlations, *t* tests, and Mann–Whitney *U* tests as appropriate to identify relevant confounders of cortisol (i.e. place of collection, day of collection, time of awakening, BMI, smoking status, comorbidities with other mental disorders, physical illnesses, and the intake of medication). No significant confounders of cortisol were found and thus *t* tests were used for group comparisons between TRUD and healthy controls, TRBD and healthy controls, and TRUD vs. TRBD. For non-parametric data, Mann–Whitney *U* tests were used. All reported *p* values are two-tailed and a correction for multiple comparisons was applied using the rough false discovery rate^27^. Data were analysed using the Statistical Package for Social Sciences (SPSS).

## Results

### Sample characteristics

Demographics of all participants are shown in Table 1. Our sample mainly consisted of middle-aged adults, with a female preponderance. Three bipolar and two unipolar patients had been seen only as outpatients; the rest had been inpatients. According to the BMI, both patients and controls were overweight on average. In both patient groups, comorbidity rates with anxiety disorders were highest. The large majority of patients were taking medication at the time of assessment, most commonly antidepressants and mood stabilisers. In terms of part treatment history, patients were highly treatment resistant, with mostly around ten failed treatments.

**Table 1:** Characteristics of patients with treatment-resistant unipolar depression, treatment-resistant bipolar depression, and healthy controls.

	TRUD (*n* = 37) Mean ± SD, *n* (%)	TRBD (*n* = 17) Mean ± SD, *n* (%)	HC (*n* = 47) Mean ± SD, *n* (%)
Age (years)	52 ± 13	52 ± 10	50 ± 16
Sex			
Male	9 (24%)	7 (41%)	21 (45%)
Female	28 (76%)	10 (59%)	26 (55%)
BMI (kg/m²)	30 ± 7.3	27.5 ± 6.2	26.3 ± 4.5
Duration of current episode (years), median (IQR)	4 (5)	3 (3)	−
Total illness duration (years), median (IQR)	13 (19)	15 (13)	
Previous treatment failures, median (IQR)			
Antidepressants	6 (3)	4 (4)	
Mood stabilisers	2 (1)	3 (2)	
Antipsychotics	1 (2)	1 (1)	
Augmenters	0 (1)	0 (1)	
Anxiolytics/hypnotics	1 (1)	0 (1)	
All medication	11 (7)	9 (7)	
Previous ECT^a^	18/30 (60%)	6/15 (40%)	
Comorbidity			−
Anxiety disorder	17 (46%)	5 (29%)	
Eating disorders	2 (5%)	1 (6%)	
Personality disorders	4 (11%)	1 (6%)	
Medication			−
Antipsychotics	12 (32%)	1 (6%)	
Benzodiazepines/hypnotics	18 (49%)	4 (24%)	
One mood stabilisers	16 (43%)	4 (24%)	
≥ 2 mood stabilisers	5 (14%)	10 (59%)	
SNRI	12 (32%)	3 (18%)	
SSRI	6 (16%)	0 (0%)	
Tricyclics	8 (22%)	1 (6%)	
Other antidepressants	12 (32%)	2 (12%)	
Thyroid hormones	8 (22%)	3 (18%)	

*BMI* body mass index, *HC* healthy controls, *SNRI* serotonin noradrenaline reuptake inhibitors, *SSRI* selective serotonin reuptake inhibitors, *TRBD* treatment resistant bipolar depression, *TRUD* treatment resistant unipolar depression

^a^Data not available for all patients.

### Treatment resistant unipolar depression vs. healthy controls

In total, 37 patients with TRUD and a mean HAMD 21 score of 23.5 ± 5.5 were recruited into the study. The median duration of illness since the age of onset was 13 (IQR 19) years. The median duration of the current depressive episode was 4 (5) years.

**Fig. 1:**
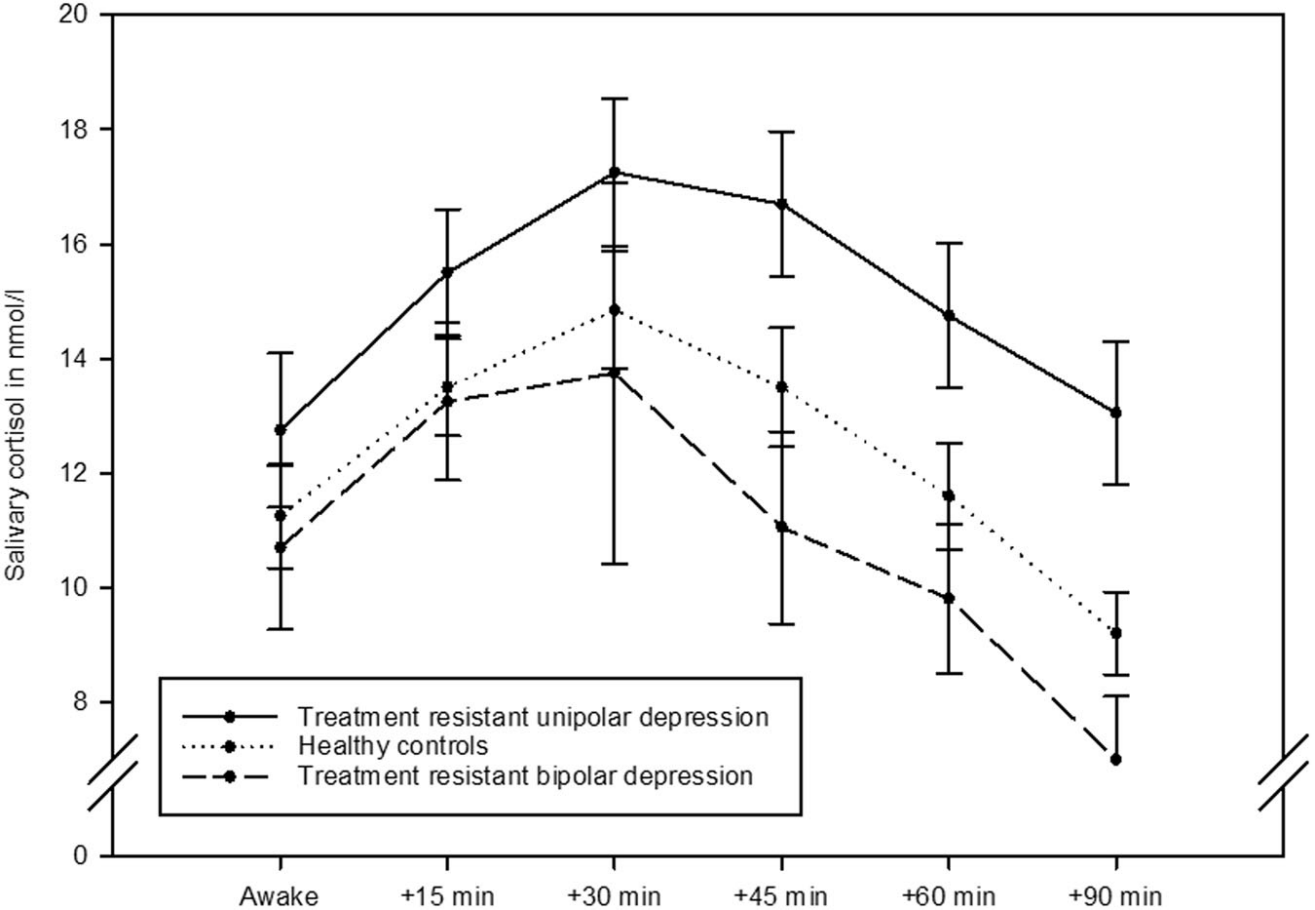
Post-awakening cortisol by group. Graph of post-awakening cortisol concentrations in patients with treatment-resistant unipolar depression (*n* = 37), treatment-resistant bipolar depression (*n* = 17), and in healthy controls (*n* = 47); means and standard errors of means, averaged across two consecutive days.

**Fig. 2:**
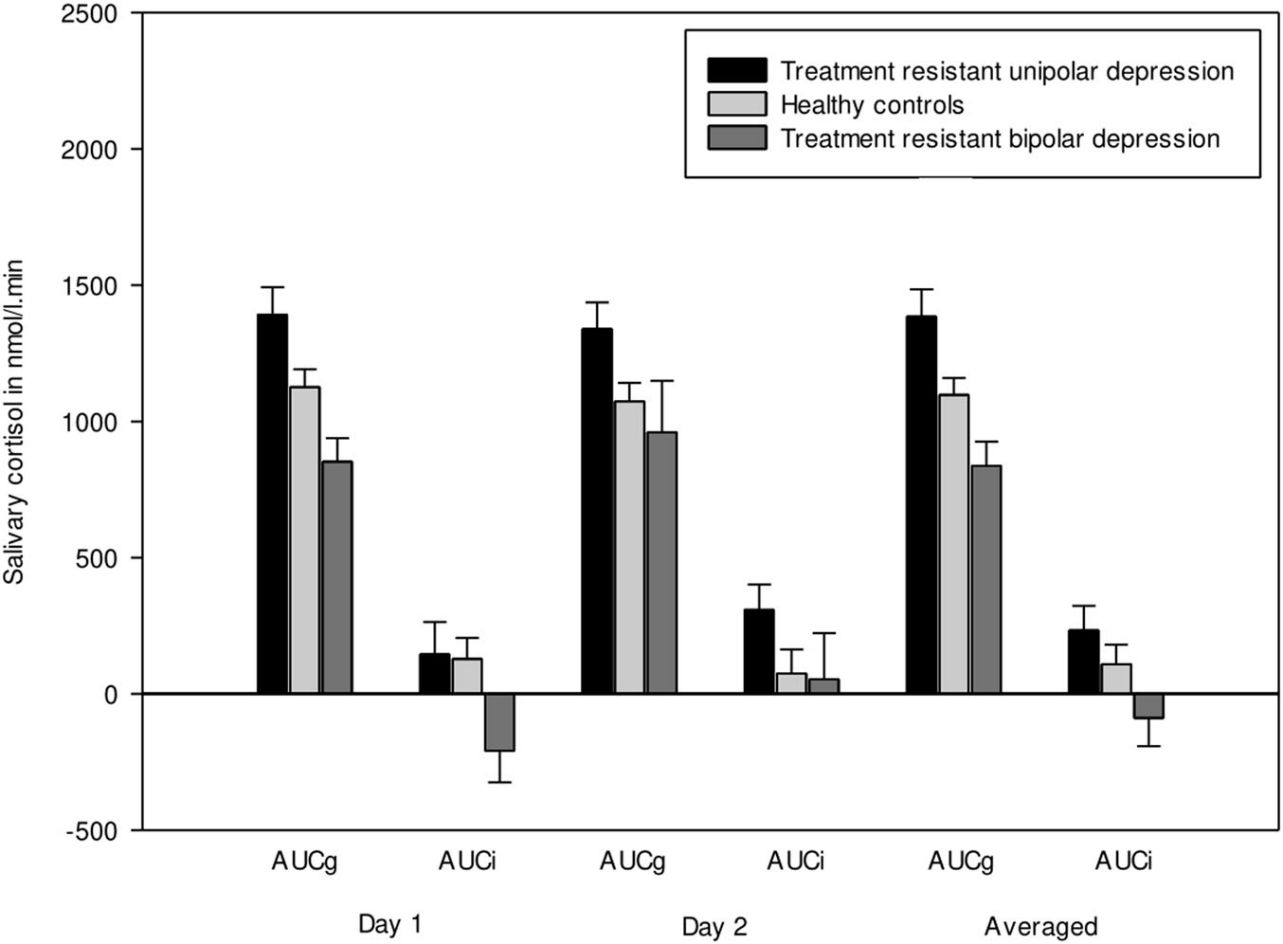
Area under the curve of post-awakening cortisol by group and by day. Bar charts of post-awakening cortisol calculated as the area under the curve in patients with treatment-resistant unipolar depression (*n* = 37), treatment-resistant bipolar depression (*n* = 17), and in healthy controls (*n* = 47); means and standard errors of means on day 1, day 2, and averaged across both days (from left to right); see manuscript for results of statistical comparison between groups.

Post-awakening cortisol concentrations of patients with TRUD at each time point are shown in Fig. 1. The averaged AUCg for the two measurement days was higher in patients with TRUD compared to controls (1385.6 ± 516.0 nmol/l.min vs. 1097.7 ± 392.0 nmol/l.min; *t* = 2.576, df = 64, *p* = 0.01). The mean AUCi, by contrast, did not differ between patients and controls (232.6 ± 468.9 nmol/l.min vs. 108.6 ± 448.9 nmol/l.min; *t* = 1.084, df = 64, *p* = 0.28). Results did not change when looking at both assessment days separately (data not shown, but see Fig. 2).

### Treatment-resistant bipolar depression vs. healthy controls

In total, 17 patients with TRBD and a mean HAMD 21 of 23.9 ± 4.5 were recruited into the study. The median duration of illness since the age of onset was 15 (IQR 13) years. The median duration of the current depressive episode was 3 (3) years.

Post-awakening cortisol concentrations in patients with TRBD at each time point are shown in Fig. 1. The averaged AUCg over the two assessment days was lower in patients compared to controls, although only by trend (836.4 (IQR 336.6) nmol/l.min vs. 1106.2 (619.1) nmol/l.min; *z* = −1.796, *p* = 0.07). The AUCi was not significantly different between patients and controls (−88.8 ± 387.2 nmol/l.min vs. 108.6 ± 448.9 nmol/l.min; *t* = −1.460, df = 51, *p* = 0.15). When looking at both assessment days individually, it became clear that the trend in the averaged AUCg was driven by a significant group difference on day 1 (patients: 852.6 ± 322.5 nmol/l.min vs. controls: 1126.0 ± 434.9 nmol/l.min; *t* = −2.165, df = 56, *p* = 0.03), whereas patients and controls did not differ on day 2 (960.0 (779.6) nmol/l.min vs. 1060.4 (682.1) nmol/l.min; *z* = −0.244, *p* = 0.80; see also Fig. 2). Similarly, groups differed in their AUCi on day 1 (patients: −209.2 (434.4) vs. controls: 184.5 (652.1); *z* = −2.017, *p* = 0.04), but not on day 2 (53.2 ± 698 vs. 74.9 ± 552.2; *t* = −0.130, df = 55, *p* = 0.90).

### Treatment-resistant unipolar depression vs. treatment-resistant bipolar depression

Post-awakening cortisol concentrations in both patient groups are displayed in Fig. 1. The averaged AUCg was higher in patients with TRUD when compared to patients with TRBD (1385.6 ± 516.0 nmol/l.min vs. 902.2 ± 304.3 nmol/l.min; *t* = 3.216, df = 39, *p* = 0.003), and the same was true regarding the AUCi (232.6 ± 468.9 nmol/l.min vs. −88.8 ± 387.2 nmol/l.min; *t* = 2.201, df = 39, *p* = 0.03). Again, these differences seemed to be more apparent on day 1 (AUCg: 1392.2 ± 559.4 nmol/l.min vs. 852.6 ± 322.5 nmol/l.min; *t* = 3.353, df = 43, *p* = 0.002; AUCi: 77.2 (861.0) nmol/l.min vs. −209.2 (434.4) nmol/l.min; *z* = 2.023, *p* = 0.04) rather than on day 2 (AUCg: 1339.5 ± 550.6 nmol/l.min vs. 1101.5 ± 527.4 nmol/l.min; *t* = 1.461, df = 47, *p* = 0.15; AUCi: 307.9 (529.6) nmol/l.min vs. 53.2 (628.0) nmol/l.min; *t* = 1.501, df = 47, *p* = 0.14).

## Discussion

The main finding of this study is that patients with TRUD had higher total cortisol concentrations in the morning hours when compared to healthy controls. In contrast, patients with TRBD exhibited a change in the opposite direction, i.e. a tendency towards diminished post-awakening cortisol concentrations. There was a clear difference in post-awakening cortisol when TRUD and TRBD were directly compared.

To the best of our knowledge, this is the first time that post-awakening cortisol concentrations were measured in clearly defined TRUD, although given that in the McAllister-Williams et al.^17^ study all patients had to use serotonergic antidepressants upon study entry, their sample of patients with a treatment resistant major depressive episode is likely to contain a high proportion of patients with TRUD. Our finding of relatively elevated post-awakening cortisol concentrations contradicts the null-finding by McAllister-Williams et al.^17^, who may have also included cases with TRBD, while being in line with previous research confined to patients with unipolar depression^24,28^. Notably, we found total cortisol production during the morning hours rather than the CAR to distinguish patients with TRUD from healthy controls. This dissociation between the AUCg and AUCi is in line with findings from the Netherlands Study of Depression and Anxiety^28^, and may mean that (morning) HPA activity rather than reactivity is abnormal in TRUD. This notion is compatible with meta-analytical evidence failing to support altered cortisol responses in depression both upon pharmacological stimulation with exogenous corticotropin-releasing hormone (CRH) or adrenocorticotropic hormone (ACTH)^4^, and upon being exposed to acute psychosocial stress^29^. Notably, Vreeburg et al.^28^ found the AUCg elevated in remitted patients as well, which may mean that elevated total morning cortisol is a trait-like marker of unipolar depression rather than being specifically linked to treatment resistance.

Contrary to TRUD, we found a tendency in patients with TRBD to show attenuated total cortisol concentrations in the morning hours. This is the first time that post-awakening concentrations were measured in patients with clearly defined TRBD. In fact, barely any research has investigated post-awakening cortisol in patients with bipolar disorder generally and bipolar depression in particular—regardless of treatment resistance^6^. This means that it remains unclear to what extent the present findings are applicable to TRBD only or to patients with bipolar disorder in general. Of the three previous studies that have measured this parameter, one found higher^9^ and two normal^7,8^ post-awakening cortisol levels in bipolar depression. Notably, all of these studies measured cortisol on one day only, whereas we averaged it over two days. This latter protocol is in line with recently published guidelines, which recommend that CAR data are obtained on at least 2 days due to their high susceptibility to the influence of state variables^30^. In addition, the study by Jabben et al.^9^ used a self-report screen for bipolar spectrum rather than a formal clinical diagnosis, and is thus likely to contain many patients not meeting full criteria for bipolar disorder.

It is noteworthy, however, that our finding was driven by significant differences between TRBD and healthy controls on assessment day 1, while the primary analyses (averaged analyses) only revealed a trend. Novelty effects could have driven the observed differences between patients and controls on the first assessment day, while preventing us from detecting significant differences on day 2, when participants were more familiar with the protocol. Although not specifically studied in relation to post-cortisol awakening cortisol concentrations, the literature on exposure to repeated acute psychosocial stress in the laboratory suggests novelty to be an essential element underlying HPA axis responses^31^. In this context, higher trait novelty seeking—which is more prevalent in bipolar disorder when compared to both unipolar disorder and healthy individuals^32^—might have amplified the herein found difference on the first assessment day by moderating post-awakening cortisol output. This would fit in well with studies showing that healthy individuals scoring high on novelty seeking demonstrated relatively diminished cortisol reactivity to different types of stimuli challenging the HPA axis^33,34^. However, as we did not assess temperament in the present study, this notion remains purely speculative. Also, the small size of the TRBD group somewhat limits the interpretation of this finding, and more research in this highly selected group of patients is warranted in the future.

We found opposite post-awakening cortisol patterns in TRUD and TRBD: whereas our TRUD group exhibited a similar cortisol pattern to that of the healthy controls, the TRBD group appeared to be characterised by a more rapid decrease in cortisol starting 30 min after awakening. This finding contradicts a previous study that did not find any differences between depressed patients with vs. without a history of a hypomanic episode^35^. The possible inclusion of bipolar spectrum disorder rather than ICD-10 or DSM bipolar disorder and the use of only one day of cortisol measurement may account for this discrepancy. Furthermore, Becking et al.^35^ included individuals with less severe forms of depression. Indeed, it would be highly interesting for future research to investigate whether the observed differences only emerge in specific subtypes of patients with (treatment-resistant) depression and whether there is a dose-response relationship between the number of depressive episodes experienced and the degree of alterations in post-awakening cortisol.

A number of limitations need to be acknowledged. First, our sample was recruited from a specialist service of treatment-resistant patients and so our results may not be encountered in other settings that attract patients with less severe depressive symptoms. Second, given that there is no widely accepted definition of TRBD, the same criteria as for TRUD were used in the present study. It might be argued that failure to respond to antidepressant medication in bipolar depression may not represent true treatment resistance, but rather a ‘paradigm failure’ in treatment^3,36^ and thus pseudo-resistance instead. Against this, whilst we defined treatment resistance according to failed antidepressant treatment in order to standardise across all patients, in fact the patients with TRBD in this study had previously also received adequate treatment trials with an average of four non-antipsychotic or antipsychotic mood stabilisers in addition to antidepressants. This represents a similar level of resistance to that seen in clinical studies of TRBD^37^ and to recent suggestions for defining TRBD that involve two failed mood stabiliser treatments^22^, and confirms the high degree of treatment resistance to targeted treatments for bipolar depression in the studied group. Moreover, the use of antidepressants in bipolar depression remains a commonly used real-world treatment option^38^. Third, using participants on medication is a limitation, given that many psychotropic medications affect the HPA axis. For instance, Sarubin et al.^39^ recently showed that both quetiapine and escitalopram exerted an effect on depressed patients’ HPA functioning when administered over 5 weeks. More specifically, quetiapine was shown to mildly dampen cortisol levels, whereas only a transient rise in cortisol was observed in the escitalopram group. Similarly, mood stabilisers, such as lithium, seem to affect the HPA axis in the long-term, such as that current intake was linked to normal or elevated cortisol concentrations in patients with bipolar disorder^40^. However, it is unlikely that medication accounted for the differences found between TRUD and TRBD, since our two samples were taking similar medications and to a similar extent, medication intake was found statistically unrelated to cortisol concentrations, and, if anything, opposite findings would have been expected based on the available literature. Fourth, the CAR has been linked to certain sleep-related variables, namely sleep duration and waking time, albeit very inconsistently^41^. While the design of the present study did not allow us to obtain polysomnographic sleep measures, awakening time was not related to post-awakening cortisol. Notwithstanding this, it would be interesting for future studies to obtain objective and/or subjective measures of sleep, and to study these in relation to depression and the CAR. We further encourage any studies interested in the CAR to adhere to the recently published expert consensus guidelines, which provide an excellent review of the methodological challenges pertaining to its measurement^30^.

The tendency towards low post-awakening cortisol concentrations in TRBD may have important clinical implications. Provided this is replicated in future studies, it would subsequently be useful to clarify whether hypocortisolaemia in TRBD is a product of treatment resistance, perhaps induced by the chronicity of the illness, or whether hypocortisolaemia instead contributes to treatment resistance. In unipolar depression, evidence is in fact accumulating for the latter, that is, for HPA axis abnormalities to be related to worse outcomes after first-line treatments^42,43^. If this proves true in bipolar depression as well, novel treatment strategies may be indicated for this patient group. In relation to pharmacological treatment, methods to increase HPA axis activity may be beneficial in TRBD as opposed to the existing strategies to decrease HPA activity, which are often used in unipolar depression, including the known effects of standard antidepressants and specific strategies to lower HPA activity such as ketoconazole or metyrapone^44^. Notably, the latter may only prove useful in a subgroup of patients with pre-treatment HPA abnormalities, as suggested by McAllister-Williams et al.^17^. The potential beneficial effects of increasing HPA axis activity may mirror the benefits seen in other conditions that may overlap with atypical depression, such as chronic fatigue syndrome^45^. It may thus be that post-awakening cortisol levels could not only serve as a biomarker that allows differentiating between unipolar and bipolar depression, but also an indicative biomarker to optimise choice of therapy. Bearing in mind the complexities attached to its measurement^30^, further research is warranted to replicate our findings and to evaluate its diagnostic and prognostic potential against other biomarkers.
